# High resolution 4D HPCH experiment for sequential assignment of ^13^C-labeled RNAs via phosphodiester backbone

**DOI:** 10.1007/s10858-015-9989-5

**Published:** 2015-09-26

**Authors:** Saurabh Saxena, Jan Stanek, Mirko Cevec, Janez Plavec, Wiktor Koźmiński

**Affiliations:** Faculty of Chemistry, Biological and Chemical Research Centre, University of Warsaw, Żwirki i Wigury 101, 02089 Warsaw, Poland; Slovenian NMR Centre, National Institute of Chemistry, 1000 Ljubljana, Slovenia; EN-FIST Centre of Excellence, 1000 Ljubljana, Slovenia; Faculty of Chemistry and Chemical Technology, University of Ljubljana, 1000 Ljubljana, Slovenia

**Keywords:** RNA resonance assignment, HCP, Selective pulses, Four-dimensional NMR, Non-uniform sampling

## Abstract

The three-dimensional structure determination of RNAs by NMR spectroscopy requires sequential resonance assignment, often hampered by assignment ambiguities and limited dispersion of ^1^H and ^13^C chemical shifts, especially of C4′/H4′. Here we present a novel through-bond 4D HPCH NMR experiment involving phosphate backbone where C4′–H4′ correlations are resolved along the ^1^H3′–^31^P spectral planes. The experiment provides high peak resolution and effectively removes ambiguities encountered during assignments. Enhanced peak dispersion is provided by the inclusion of additional ^31^P and ^1^H3′ dimensions and constant-time evolution of chemical shifts. High spectral resolution is obtained by using non-uniform sampling in three indirect dimensions. The experiment fully utilizes the isotopic ^13^C-labeling with evolution of C4′ carbons. Band selective ^13^C inversion pulses are used to achieve selectivity and prevent signal dephasing due to the C4′–C3′ and C4′–C5′ homonuclear couplings. Multiple quantum line narrowing is employed to minimize sensitivity loses. The 4D HPCH experiment is verified and successfully applied to a non-coding 34-nt RNA consisting typical structure elements and a 14-nt RNA hairpin capped by cUUCGg tetraloop.

## Introduction

Recent advances in RNA research has led to the discovery of several new classes of non-coding RNAs (e.g. siRNA, miRNAs) many of whose functions remain largely unknown. The function of RNA during normal and diseased states has long been studied through its structure–function relationship (Briones et al. 2009; Mercer et al. 2009). Various NMR approaches (Varani et al. 1996; Wijmenga and van Buuren 1998; Furtig et al. 2003; Flinders and Dieckmann 2006) have successfully been utilized in this regard which expanded our knowledge about RNAs structure. However, precise three-dimensional structure determination of even moderately sized RNAs is still a bottleneck due to spectral overlap.

Sequential assignment of chemical shifts is one of the prerequisite for RNA structure determination by NMR spectroscopy. The assignment is usually based on through-space 2/3D NOE-type (Nikonowicz and Pardi 1993) and/or through-bond 3D HCP-type (Marino et al. 1994) experiments. However, most often the efficacy of both type of approaches is severely affected by low chemical shift dispersion of the involved nuclei, which results into spectral overlaps making the unambiguous resonance assignment a challenging task. In non-coding RNAs, similar chemical shifts in helical secondary structures and frequent lack of base stacking make the spectral crowding particularly demanding. Recently, an alternate automated approach involving no isotope labeling (Aeschbacher et al. 2013) for RNAs was proposed that requires peak lists from 2D TOCSY, 2D NOESY and natural abundance ^1^H–^13^C HSQC spectra. However, owing to irregular and very limited statistical data basis, difficulty may arise while assigning certain regions of RNAs. In addition, chemical shift degeneracy or low dispersion still remain a challenge for such approaches. These difficulties can be alleviated using similar high dimensional approaches (Kazimierczuk et al. 2013; Mobli and Hoch 2014b; Nowakowski et al. 2015) that have been successfully applied to proteins (Stanek et al. 2013a, c; Geist et al. 2013a, b; Bermel et al. 2013). A high-resolution 4D C_aro_,C_ribo_-NOESY experiment (Stanek et al. 2013b) was recently reported which provides intra and inter-nucleotide NOEs in RNA. Since NOE interactions have conformation dependencies, ambiguities and missing links may still arise during assignments via NOESY spectrum. One approach to find such missing links in NOESY spectrum is by retrieving complementary information from some through-bond correlations. To address this issue we recently reported a high-resolution 4D HC(P)CH experiment (Saxena et al. 2014) that provides sequential connectivities through linking the neighbouring H4′–C4′ planes. Although the experiment performs well for the regions having stem and bulges in non-coding RNAs, it faces difficulties with internal- and hairpin-loop regions. As the experiment involves two long transfer periods (C4′ → P and P → C4′), it significantly relies on C4′–P (^3^*J*) couplings, which are usually weaker for such regions (Schwalbe et al. 1994; Legault et al. 1995; Hu et al. 1999), an efficient coherence transfer could become an issue. Combined with long ^13^C–^31^P coupling-refocusing and coupling-evolution delays on phosphorous nuclei the NMR signal linking aforementioned regions could have low S/N ratio. It is evident that similar approaches involving more favourable couplings and shorter delays can be quite beneficial.

An alternative, but somewhat underutilized approach is to achieve sequential H3′_i−1_–P_i_ connectivities via H3′–P couplings (Sklenář et al. 1986; Kellogg 1992; Varani et al. 1995). The reasonably favourable ^1^H–^31^P (^3^*J*) couplings (~7 Hz) can be utilized to directly transfer the initial coherence to phosphorous nuclei and enable sequential assignment via backbone. With recent advancement in high dimensional NMR techniques (Kazimierczuk et al. 2010, 2013; Mobli and Hoch 2014a) this approach can be effectively extended to moderately sized RNAs.

Here we report a high resolution C4′/H4′ selective 4D HPCH experiment which includes the chemical shifts evolution of ^1^H3′, ^31^P, ^13^C4′ and ^1^H4′ of adjoining nucleotides thereby linking them in a single experiment providing higher peak resolution. The relatively better dispersed H3′ chemical shifts aided by additional P dimension and constant time evolution of all indirect dimensions collectively facilitated to resolve poorly overlapped C4′–H4′ region. Compared to 3D HCP and 4D HC(P)CH, which initially use two coherence transfer steps (H4′ → C4′ and then C4′ → P), 4D HPCH use only one step to transfer the coherence onto phosphorous nuclei (H3′ → P) using ^1^H–^31^P (^3^*J*) couplings. The proposed 4D HPCH experiment provides sequential connectivities via H3′_i−1_–P_i_–C4′_i_–H4′_i_ links. For the cases where H5′i–Pi couplings are not weak, an additional peak giving rise to H5′_i_–P_i_–C4′_i−1_–H4′_i−1_ links further facilitates the sequential assignment. We implemented the non-uniform sampling (NUS) to achieve high resolution in all indirect dimensions. The experiment efficiently facilitates the assignment of sequential links in a non-coding RNA including the internal- and hairpin-looped regions.

## Materials and methods

### RNA samples

Two RNA samples are used to test and verify the efficacy of 4D HPCH experiment. The 14-nt cUUCGg-hairpin (5′-GGCAC(**UUCG**)GUGCC-3′; bold residues indicate the tetraloop-residues) was purchased from Silantes GmbH (Munich, Germany) as a uniformly ^13^C,^15^N-labeled RNA. The concentration of the NMR sample was 0.7 mM in 20 mM KH_2_PO_4_/K_2_HPO_4_, pH 6.4, 0.4 mM EDTA in D_2_O. Second RNA sample was ^13^C,^15^N-labeled 34-nt hairpin RNA *LCS1co* consisting of two A-RNA form stems, one adenine bulge, an asymmetric internal loop and a GAAA terminal loop (Cevec et al. 2010). RNA sample was prepared by in vitro transcription with T7 RNA polymerase (Promega), purified by denaturating PAGE, electroeluted and dialyzed against an NMR buffer. The concentration of RNA sample was 1.5 mM in 10 mM sodium phosphate buffer, pH 6.8, 20 mM NaCl in D_2_O.

### Experimental

**Fig. 1:**
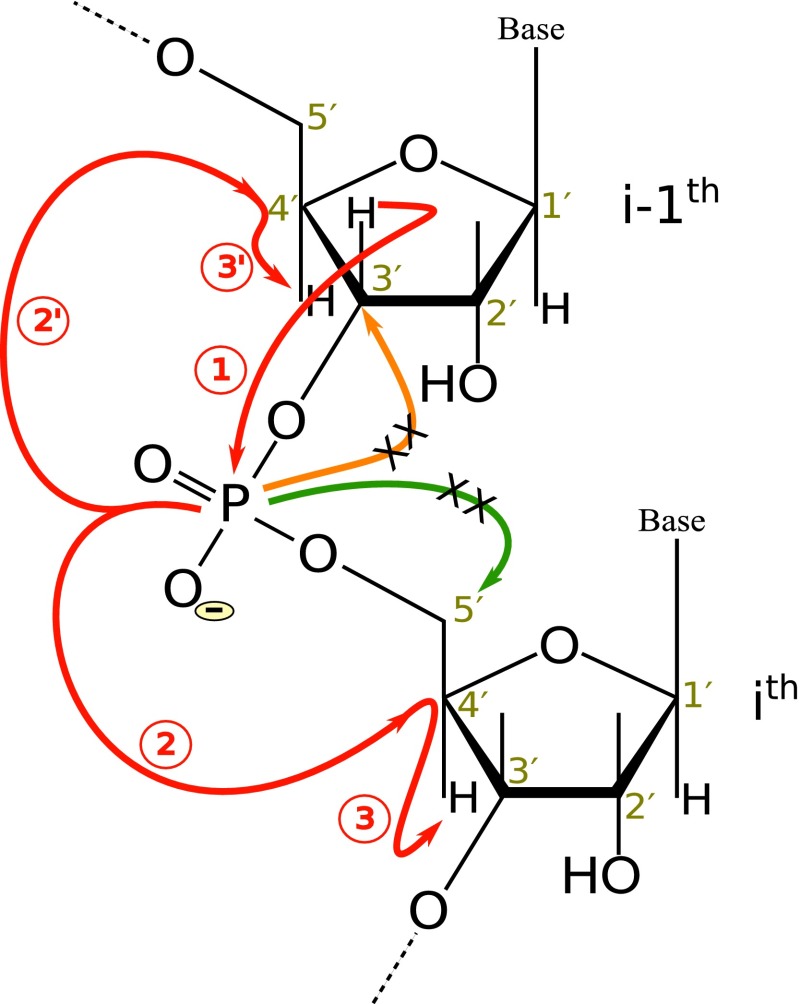
A schematic illustration of magnetization transfer in C4′/H4′ selective 4D HPCH experiment. The *numbers in circles* represent the coherence transfer steps leading to intra- and inter-correlation peaks. In the standard 3D HCP experiment the magnetization when reaches on ^31^P is further transferred (P → C3′/C5′) to other sugar carbons (C3′ and C5′) whereas in 4D HPCH experiment such pathways are blocked (denoted with *cross on orange/green arrows*) to minimize the sensitivity losses. For the cases where ^3^ *J*_H5′P_ is large, the H5′i → Pi transfer is also active giving additional peak for sequential assignment, it is however not shown here for sake of clarity

All the NMR experiments were carried out on a 600 MHz Agilent DDR2 spectrometer equipped with a room-temperature “Penta” (^1^H/^13^C/^15^N/^2^H/^31^P) probe. The experiments were acquired at a temperature of 298 K. The coherence transfer pathway scheme for 4D HPCH experiment is shown in Fig. 1. The experiment is designed with an emphasis on achieving higher resolution with minimum sensitivity losses. High dimensionality is achieved by incorporating three indirect chemical shift evolution periods into the sequence. Multiple quantum (MQ) line narrowing effect (Marino et al. 1997; Fiala et al. 1998) is utilized for slower relaxation and improve the sensitivity of the experiment. In addition, the absence of an additional C4′–P refocusing delay significantly enhanced the sensitivity compared to 4D HC(P)CH experiment reported previously (Saxena et al. 2014). The experiment utilizes the C3′/C5′ selective inversion pulses to prevent signal modulation due to ^13^C–^13^C homonuclear couplings; these pulses also indirectly enforce the C4′/H4′ selectivity and further enhance the sensitivity.

**Fig. 2:**
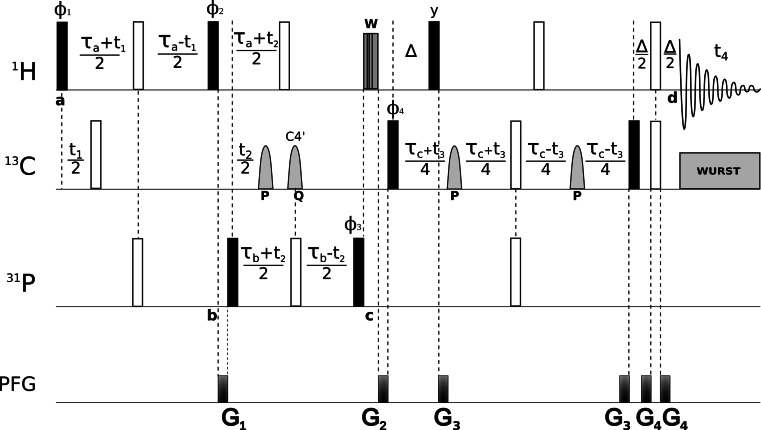
Pulse sequence scheme for through-bond, C4′/H4′ selective 4D HPCH experiment. The 90° and 180° ‘hard’ pulses are represented by *filled and open bars*, respectively. All pulses are applied along the x-axis of the rotating frame unless indicated otherwise. *Grey* sine bell-shaped pulses (**P** and **Q**) indicate cosine modulated IBURP-2 (Geen and Freeman 1991) pulses. **P** inverts the ^13^C spins in the chemical shift range of 69.5 ± 6 ppm (C3′s and C5′s) with a duration of 2.5 ms (1.9 kHz peak r.f. field) and **Q** inverts the ^13^C spins in chemical shift range 83 ± 8 ppm (C4′s) with a duration of 1.9 ms (2.5 kHz peak r.f. field). **W** represents spin-lock pulses [r.f. power 7.6 kHz, length 7.02 ms (SL_x_), 3.90 ms (SL_y_)] implemented for dephasing of transverse water magnetization. ^13^C adiabatic composite pulse decoupling was performed with WURST (Kupce and Freeman 1995). The durations of ‘hard’ *π/*2 pulses were 7.8, 18.1 and 26.5 µs for ^1^H, ^13^C and ^31^P, respectively. Proton carrier frequency was set on resonance with water (4.68 ppm), carbon carrier was set to the center of ^13^C4′s (83.00 ppm) and ^31^P carrier was set to −4.10 ppm. Quadrature detection in *t*_1_, *t*_2_ and *t*_3_ is accomplished by altering ϕ_1_, ϕ_3_ and ϕ_4_, respectively, according to the States-TPPI procedure. 8-step phase cycle is as follows: ϕ_1_ = *x*; ϕ_2_ = *y*, −*y*; ϕ_3_ = 2(*x*), 2(−*x*); ϕ_4_ = 4(*x*), 4(−*x*) and ϕ_rec_ = *y*, 2(−*y*), *y*, −*y*, 2(*y*), −*y*. Delays are set as follows: ∆ = 3.5 ms ≈ (2 *J*_CH_)^−1^, τ_a_ = 19 ms, τ_b_ = 21 ms and τ_c_ = 20.9 ms. Gradient levels and durations are: G_1_ (0.5 ms, 21.7 G/cm), G_2_ (0.8 ms, 34.2 G/cm), G_3_ (0.2 ms, 17.5 G/cm) and G_4_ (0.5 ms, 12.61 G/cm). A total of 2350 (~18 %) sampling points (*t*_1_, *t*_2_, *t*_3_) were randomly chosen from a 27 × 22 × 21 Cartesian grid with uniform sampling distribution. Maximum evolution times of 18 (*t*_1*max*_), 18 (*t*_2*max*_) and 14 ms (*t*_3*max*_) were achieved in the indirectly detected dimensions. Acquisition time was set to 85 ms (*t*_4*max*_). Spectral widths of 15 (ω_1_), 12 (ω_2_), 15 (ω_3_) and 12 kHz (ω_4_) were assumed. The total experiment duration was 75 h. The interscan delay of 1.6 s for optimal recovery of ^1^H magnetization (sensitivity per unit time) was used. The experiment was performed at 298 K on the Agilent DDR2 600 MHz spectrometer equipped with a room-temperature “Penta” (^1^H/^13^C/^15^N/^2^H/^31^P) probe

The pulse sequence (see Fig. 2) comprises ^1^H to ^31^P transfer period, a middle ^31^P–^13^C single quantum transfer period and one ^1^H–^13^C MQ period. In the first period, the coherence originates from H3′ sugar protons (owing to the reasonably large ^3^J_H3′,P_ (> ^3^J_H5′,P_) couplings) and is transferred to ^31^P via INEPT. For the cases where H5′–P couplings are not weak, the H5′/5″ → P coherence transfer is also involved which provides H5′_i_–P_i_–C4′_i−1_–H4′_i−1_ sequential links. During same period the chemical shifts of H3′ (H5′) protons are evolved in a constant time manner (*t*_1_). In order to refocus undesired evolution of ^1^H–^13^C couplings a 180° pulse on ^13^C channel is applied. A crush-gradient is applied afterward to remove the coherences that do not follow the desired coherence pathway. The subsequent 90° pulses on ^1^H and ^31^P transfer the coherence onto ^31^P transverse plane, where its chemical shift is also evolved (*t*_2_) in a constant time. During this period a pair of cosine modulated (see Fig. 2 caption for details) IBURP-2 (Geen and Freeman 1991) pulses are used to achieve selectivity and reduce sensitivity losses. Here the ^31^P–^13^C4′ couplings are evolved, and a C3′/5′ selective cosine modulated IBURP-2 pulse (P in Fig. 2) is employed to prevent dephasing due to P_i_–C3′_i−1_ and P_i_–C5′_i_ couplings. Also, to achieve selectivity for P → C4′ transfer, C4′ selective cosine modulated IBURP-2 pulse (Q in Fig. 2) is used simultaneously with the 180° pulse on ^31^P. A 180° pulse on ^1^H is applied to refocus the P_i_–H_i−1_ anti-phase coherence created during previous period. The coupling delays ideally required (71 and 62 ms for H3′ → P and P → C4′ transfer, respectively) for a complete transfer were optimized with relaxation taken into account. The compromised final delays were set to 19 and 21 ms for H3′ → P and P → C4′ transfer, respectively.

In the consecutive block, the coherence is forward transferred to C4′_i_ where its chemical shifts are indirectly recorded (*t*_3_). Since, as it was shown earlier for nucleic acids (Fiala et al. 1998; Fiala et al. 2000), the dominant ^1^H–^13^C dipolar relaxation mechanism is significantly attenuated for zero- and double-quantum coherences, MQ coherences are preserved during the frequency labeling of C4′ evolution period. During the same period (τ_c_) refocusing of P–C4′ anti-phase coherence is also achieved by application of a 180° pulse on ^31^P in synchrony with the moving 180° pulse on ^13^C channel. Another 180° pulse is centrally placed on ^1^H channel to refocus its chemical shift evolution. During this period, the evolution due to homonuclear carbon coupling (C4′–C3′ and C4′–C5′) is refocused by two cosine modulated IBURP-2 (Geen and Freeman 1991) pulses (P in Fig. 2) which selectively and simultaneously invert the frequency bands of C3′ and C5′ ribose sugar carbons. Since C2′ carbons share the same spectral region as of C3′, inversion of later also inverts the C2′ carbons. Effectively, the use of inversion pulses leads to an indirect selection of C4′ and hence H4′ during this period. Finally, an in-phase coherence is generated on H4′ spins by refocused INEPT transfer during ∆ period.

The inversion profiles for shaped pulses are simulated and tested using Spinach library (Hogben et al. 2011) on MATLAB^®^. Gradients and phase cycling are employed to eliminate undesired coherences and improve the C4′/H4′ selectivity of experiment. After P → C4′ transfer period, spin-lock pulses (SL_x_, SL_y_) are employed (W in Fig. 2) to dephase any remaining transverse water magnetization. To achieve higher dimensionality with reasonable resolution in the indirectly detected dimensions, non-uniform sampling was employed. Using NUS we are able to acquire 4D HPCH experiment with high evolution times: 18 ms (*t*_1_), 18 ms (*t*_2_) and 14 ms (*t*_3_).

The processing of 4D NUS data was accomplished by the home-written software package SSA (Signal Separation Algorithm) (Stanek et al. 2012), which can be downloaded free of charge for non-commercial purposes from the website http://nmr.cent3.uw.edu.pl.

## Results and discussion

**Fig. 3:**
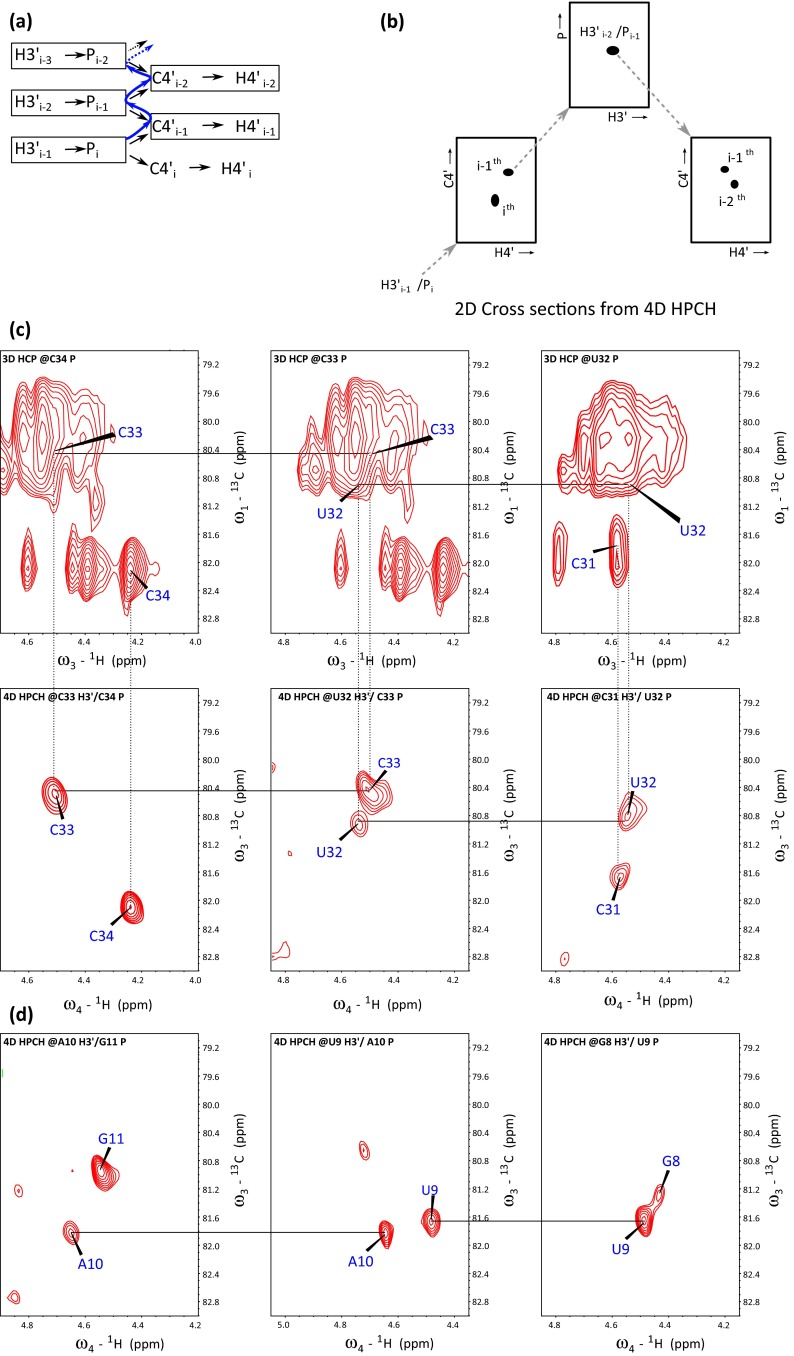
Sequential correlation by 4D HPCH experiment. **a** Illustration of coherence transfer (*black arrows*) and how sequential assignment (*blue path*) is achieved in RNA backbone using 4D HPCH experiment. **b** Schematic representation of spectral analysis and sequential assignment of peaks on 2D cross sections obtained from 4D HPCH spectrum. For the cases where H5′i–Pi couplings are not weak, second peak giving rise to H5′_i_–P_i_–C4′_i−1_–H4′_i−1_ sequential links can also be used for assignment. For the sake of clarity, only one peak is shown in H–P plane. **c** Panels on the top show overlapped H4′/C4′ region from 3D HCP spectrum fixed at ^31^P chemical shifts of 34-nt RNA *LCS1co*. Resolution enhancement can be seen in the bottom **c**, **d** panels, which are the 2D cross-sections of 4D HPCH spectrum extracted along the H3′_i−1_–P_i_ chemical shifts. The peaks in 4D HPCH spectrum are well resolved and the assignment of sequential links is achieved successfully in 34-nt RNA. Among assigned connectivities are also included the links which are present within internal- and hairpin-loops

We tested the performance of 4D HPCH experiment on a 14-nt tetra-loop RNA sample and a fairly demanding 34-nt RNA sample which encompasses typical structural elements. Figure 3c shows the representative 2D C4′–H4′ correlation planes from the 3D HCP and 4D HPCH spectra of 34-nt RNA *LCS1co* illustrating the resolution advantage in later. It can clearly be seen that, in 4D HPCH the C4′–H4′ correlation peaks have been unambiguously resolved along the additional H3′ and P dimensions or H3′–P planes (e.g. peaks correlating C34–C33, C33–U32 and U32–C31 residues). For 3D HCP the ^31^P chemical shifts evolve in real time which results in higher sensitivity but poor resolution and linewitdths (~110 and ~82 Hz for P and C4′ respectively). Noteworthy is that in 4D HPCH, chemical shifts evolution of all indirectly detected dimensions was implemented in constant time manner, providing better resolution [~40 Hz (H3′), 45 Hz (P) and ~42 Hz (C4′/H4′)]. With NUS it was possible to acquire the experiment with high evolution times which could not have been practically achieved by full grid uniform sampling.

The 4D HPCH spectrum can be easily analyzed with any NMR assignment programme. For this study we used SPARKY (Goddard and Kneller 2004) program and spectrum was analyzed by fixing two dimensions (H3′_i−1_ and P_i_) of corresponding nucleotides (for illustration see Fig. 3a and b). The resulting 2D C4′–H4′ plane consists of intra- and inter-nucleotide peaks, i.e. to the (i − 1)th and ith residue. Consecutively, (i − l)th C4′/H4′ are fixed and H3′_i−2_–P_i−1_ peak is assigned on H3′–P plane (which provides sequential link to previous nucleotide via H3′_i−2_ protons (see Fig. 3b). In earlier approaches, 3D HCP and 4D HC(P)CH, the coherence transfer efficiencies vary largely with C4′–P couplings and, in turn, with the β/ε torsional angles in different regions in RNA. For the regions where the C4′ → P couplings are weak, an efficient coherence transfer is difficult to achieve. In the presented method, however, coherence transfer is initiated by utilizing H3′–P couplings (^3^*J*_C4′,P_) which are comparatively large for aforementioned regions. This approach partially compensated the coherence transfer efficiency loss and assignment of correlation peaks belonging to such regions was achieved. For the cases which are not sensitivity limited, the proposed experiment can provide nearly complete assignment within the regions involving internal/hairpin loops in RNA. For 14-nt cUUCGg-tetraloop RNA used in this study assignment 11 out of 13 sequential peaks, including the connectivities present within cUUCGg hairpin loop, was achieved using 4D HPCH spectrum. The two missing correlations were due to small couplings (U6–U7) and heterogeneity at the 5′-end (G1–G2).

**Fig. 4:**
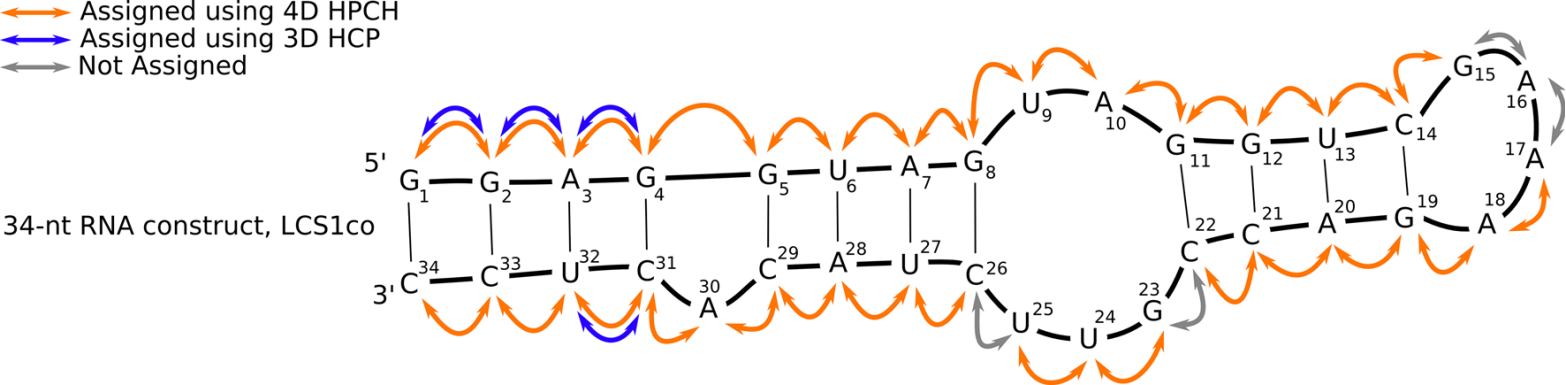
The schematic representation of the investigated 34-nt RNA showing the sequential connectivities assigned in the 3D HCP and 4D HCPCH spectrum. *Blue arrows* indicate the sequential correlations unambiguously assigned using 3D HCP experiment, while *orange arrows* indicate sequential connectivities obtained from C4′/H4′ selective 4D HPCH experiment. Non assigned correlations are marked with the *grey arrows*

For 34-nt RNA *LCS1co*, overall 29 sequential connectivities were successfully established (see Fig. 4) using 4D HPCH, whereas 3D HCP experiment could provide only 4 such sequential links unambiguously in the same experimental acquisition time. Comparatively, the previously reported 4D C_aro_,C_ribo_-NOESY and 4D HC(P)CH experiments provided 17 and 19 sequential links respectively, which reflects the difficulty of the investigated RNA sample. It is to be noted that among the assigned correlations are included some of the residues that belong to internal/hairpin loops and bulges (e.g. G8–U9, A10–G11, A17–A18, G23–U24, U24–U25 and C29–A30) which were notoriously difficult to assign by previously reported methods. Interestingly, 4D HPCH and 4D NOESY experiments provide complementary data for sequential assignment. The four missing assignments are either due to structural mobility, manifesting in enhanced relaxation during coherence transfer periods or due to weaker H3′–P/C4′–P couplings. Nevertheless, with 4D HPCH experiment, along with stem regions, we were able to assign most of the non-stem regions (bulges, internal- and hairpin-loops) in moderately sized RNA. The experiment complements the set of recently reported high dimensional experiments, 4D HC(P)CH, 5D HCP-CCH COSY (Krahenbuhl et al. 2014), 4D-NUS C_aro_,C_ribo_-NOESY (Stanek et al. 2013b) and 4D HC(P)CH (Saxena et al. 2014), dedicated for sequential resonance assignment in RNAs.

## Conclusions

In summary, we have presented a novel NMR pulse sequence for sequential assignment in RNAs via highly-resolved C4′–H4′ correlation. The sequential peaks were effectively resolved along the intranucleotide H3′–P planes. The experiment ideally complement the 4D HC(P)CH experiment by relying on different coupling constants for magnetization transfer to generate uniformly intense cross peaks in different regions of the RNA spectrum. The attenuated relaxation due to MQ coherences and C4′/H4′ selectivity in the experiment partially compensated the sensitivity losses entailing the increased dimensionality. Band selective inversion pulses were used to prevent signal modulation due to C4′–C3′, C4′–C5′ couplings and to indirectly select the C4′/H4′ region. Employing high dimensionality and non-uniform sampling, the novel method allows for the assignment of most of the sequential links in highly overlapped NMR spectra of RNAs. Given the outstanding sensitivity and spectral resolution of the NMR approach, we envisage useful applications for the assignment of hairpin/internal loop and bulge regions in RNAs. The comparative study with other experiments suggests that the presented experiment can be especially beneficial for assigning such regions in RNA. Despite lower sensitivity, the proposed experiment clearly outperforms the conventional sequential correlation approaches for RNAs which suffer from critical spectra overlap. The experiment is proposed as a complementary tool to 3D HCP, 3D/4D NOESY experiments and augments the set of high dimensional experiments aimed at improving resolution and reducing ambiguities during resonance assignments in RNAs with poor chemical shift dispersion.
